# Transcriptomic comparison between populations selected for higher and lower mobility in the red flour beetle *Tribolium castaneum*

**DOI:** 10.1038/s41598-023-50923-6

**Published:** 2024-01-02

**Authors:** Kentarou Matsumura, Takafumi Onuma, Shinji Kondo, Hideki Noguchi, Hironobu Uchiyama, Shunsuke Yajima, Ken Sasaki, Takahisa Miyatake

**Affiliations:** 1 Graduate School of Environment, Life, Natural Science and Technology, Okayama University, Okayama, Japan; 2 Graduate School of Agriculture, Tamagawa University, Tokyo, Japan; 3 Center for Genome Informatics, Research Organization of Information and Systems, Joint Support-Center for Data Science Research, 1111 Yata, Mishima, Shizuoka 411-8540 Japan; 4 NODAI Genome Research Center, Tokyo University of Agriculture, 1-1-1 Sakuragaoka, Setagaya-ku, Tokyo, 156-8502 Japan; 5 Department of Bioscience, Tokyo University of Agriculture, 1-1-1 Sakuragaoka, Setagaya-ku, Tokyo, 156-8502 Japan

**Keywords:** Ecology, Evolution, Genetics, Molecular biology, Neuroscience, Physiology

## Abstract

Movement is an important behavior observed in a wide range of taxa. Previous studies have examined genes controlling movement using wing polymorphic insects and genes controlling wing size. However, few studies have investigated genes controlling movement activity rather than morphological traits. In the present study, we conducted RNA sequencing using populations with higher (WL) and lower (WS) mobility established by artificial selection in the red flour beetle *Tribolium castaneum* and compared gene expression levels between selected populations with two replicate lines. As a result, we found significant differences between the selected populations in 677 genes expressed in one replicate line and 1198 genes expressed in another replicate line, of which 311 genes were common to the two replicate lines. Furthermore, quantitative PCR focusing on 6 of these genes revealed that neuropeptide F receptor gene (*NpF*) was significantly more highly expressed in the WL population than in the WS population, which was common to the two replicate lines. We discuss differences in genes controlling movement between walking activity and wing polymorphism.

## Introduction

Movement is an important event in the evolution of organisms because it is observed across a wide range of taxa, in both plants and animals, and leads to gene flow and the acquisition of new resources^1,2^. On the other hand, individual differences in movement are often found within populations^3–6^. Individual differences in movement suggest that there are also costs of movement. For example, because movement involves energy costs, the amount of investment in reproduction may be reduced by movement^3^. Indeed, it has been reported that movement and reproduction trade off in various species^3,7^. Thus, if the individual differences in movement evolve by natural selection, it is predicted that there are genetic bases for their differences.

In investigating the genes controlling movement, it is important to examine genes for higher and lower moving activity within species. Several previous studies have focused on genes, mostly in wing polymorphic insects. Wing polymorphic insects are better suited to uncovering the genetic basis for controlling wing size because of the presence of individuals with large and small wing sizes within the species^8^. In the stonefly *Zelandoperla fenestrata*, RNA sequencing was used to compare gene expression levels among different wing types to determine which genes control large and small wing sizes^8^. Similarly, genes controlling wing dimorphism have been investigated in aphids^9,10^ and planthoppers^11^. Much of the previous research aimed at searching for genes controlling flight dispersal in this way has involved genetic analysis of flight–insect wing polymorphisms and long-distance dispersal. On the other hand, dispersal is considered to be part of life history because it is closely related to the time of development and reproduction^5,12^. Because wing size in wing polymorphic insects is closely related to dispersal, the results of previous studies using wing polymorphic insects may not reflect general movement, such as walking movement. Thus, the selection pressures for general movement and dispersal may be very different, and their genetic bases may be different^12^. Because insects with wing polymorphisms targeted in previous studies were dispersing rather than exhibiting general movement, genes controlling general movement should also be sought, but few studies have done so.

**Figure 1:**
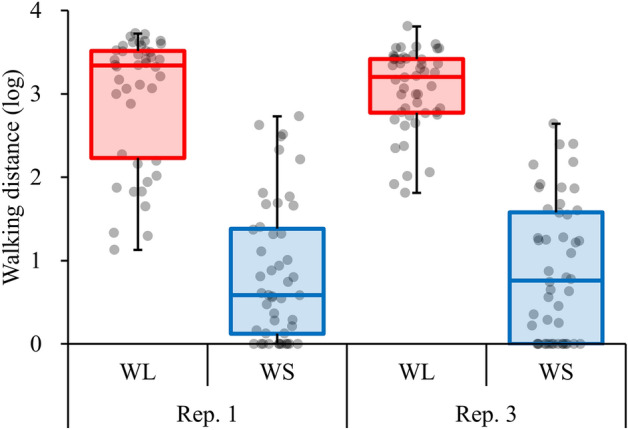
Box plot for walking distance of *T. castaneum* females from the WL and WS selection regimes in each replicate line (rep. 1 and rep. 3).

In previous studies^13–15^, we performed artificial selection for walking distance in the red flour beetle *Tribolium castaneum* and established populations with genetically determined long (WL) and short (WS) walking distances (Fig. 1). By comparing gene expression levels among selected populations, it is possible to investigate which genes govern the general movement of this species. Because its entire genome has been sequenced^16^, the flour beetle is well-suited for organisms for conducting molecular biological studies^17–20^. In a previous study, the genes that control wing development in *T. castaneum* were investigated^16^. Furthermore, although a recent study conducted artificial selection and simulation to investigate the inheritance controlling dispersal behavior in *T. castaneum*^21^, the genes that control mobility, which is a behavior rather than a morphological feature, remain unclear. Therefore, in this study, we performed RNA sequence analysis using selected populations of *T. castaneum* with different mobilities to compare gene expression levels among selected populations and to search for candidate genes controlling the general movement of this species.

## Materials and methods

### Insects

The red flour beetle, *Tribolium castaneum* (Herbst, 1797), is a stored-product insect found worldwide and a model species for genomics. The *T. castaneum* beetle culture used in this study has been maintained in the laboratory for more than 40 years^22^ according to the rearing method described by a previous study^22^. The *T. castaneum* population used in this study was also used as the ancestor of the selected populations in the previous study^22^, and RNA sequencing and DNA resequencing used the selected populations were conducted in these previous studies^19,20^. The protocol for artificial selection for mobility was described in Matsumura and Miyatake^13^. Briefly, the walking distance was measured in 75 male and 75 female adult beetles that were randomly collected from the stock population. From the population, 10 males and 10 females with the shortest and longest walking distances were allowed to reproduce for the next generation, and populations with genetically determined short (WS) and long (WL) walking distances were established, respectively. The selection regime was continued for more than 25 generations^13,15^. To increase the reproducibility of the present results, we used replicate lines that were created simultaneously during the artificial selection experiment^13^. Although three replicate lines were created under the selection regime, two replicate lines, (1) and (3)^13^, whose responses to artificial selection were clearer, were used in this study.

### RNA extraction and cDNA library preparation

We used only female beetles (adults one month after emergence) for RNA-sequencing (RNA-seq) because we wanted to eliminate the influence of sex. Female individuals from two replicates of the WS (WS1 and WS3) or WL (WL1 and WL3) populations were placed in an Eppendorf tube containing liquid nitrogen for storage. Abdomens and legs were removed from the frozen bodies using a pair of fine spring scissors on a cooling plate under a stereomicroscope, as the abdomen sometimes included items consumed by the beetle or microorganisms. We used the head and thoracic parts of one beetle per sample for RNA-seq and two beetles per sample for real-time quantitative polymerase chain reaction (RT‒qPCR). Before RNA extraction, we washed the head and thoracic parts with sterile 0.1 M phosphate buffer (pH 7.0) and then minced the tissue by a pair of sterile scissors in the extraction solution using ISOGEN (Nippongene, Tokyo, Japan) according to the manufacturer’s instructions. The extracted RNA was digested with DNase (RT Grade for Heat stop, Nippongene) at 37 °C for 15 min to remove DNA and then mixed with stop solution at 70 °C for 10 min. The extracted RNA was qualitatively and quantitatively examined at 230, 260, and 280 nm by a spectrophotometer (NanodropTM 2000, Thermo Fisher Scientific, Waltham, MA, USA), and RNA integrity was determined by using the Agilent RNA 6000 Nano Kit (Agilent Technologies) and an Agilent Bioanalyzer 2100 (Agilent Technologies, Santa Clara, CA, USA). Three samples of RNA in each replicate from two populations were selected from each of the fifteen RNA samples for RNA-seq.

### cDNA library preparation and RNA-seq

From 500 ng of the total RNAs, we purified mRNA by using the NEBNext Poly(A) mRNA Magnetic Isolation Module (New England BioLabs), and constructed the RNA-seq libraries using the NEBNext Ultra RNA Library Prep Kit for Illumina (New England BioLabs), according to the manufacturer’s protocol. Library concentration and quality were assessed with the Agilent DNA 1000 Kit using an Agilent Bioanalyzer 2100 (Agilent Technologies). In addition, the library concentration was precisely determined using the KAPA Library Quantification Kit (Kapa Biosystems, Wilmington, MA, USA) and a Step-One-Plus real-time PCR system (Applied Biosystems Laboratories, Foster City, CA, USA).

We sequenced the cDNA library on the Illumina NextSeq 500 platform (Illumina) using a flow cell with a 300-cycle NextSeq 500 Reagent Kit v2 (2 × 150-mer) (Illumina) and producing 300-bp paired reads. Reads were generated in FASTQ format using the conversion software bcl2fastq2 (Illumina, version 2.18). We submitted the read data to the Read Archive of DDBJ (accession number DRA017158).

### Analysis of differentially expressed genes (DEGs)

Using TagDust (version 1.13), P5 and P7 adaptor sequences containing raw reads were removed. In addition, we used FASTX-Toolkit, ver. 0.0.13.2, to clip and trim the first 13 bp of each read and to exclude low-quality reads. We used the following quality-filtering parameters: (i) minimum quality score, 20, and (ii) minimum percentage of bases with [− q] quality, 80. Then, the clean-read data were mapped using TopHat2 (version 2.0.12) with Bowtie2 to the reference genome of *T. castaneum* (Tcas5.2) that was obtained from the NCBI genome database (https://www.ncbi.nlm/nih.gov/). The expression level of each transcript was calculated by normalization to “fragments per kilobase of exon per million reads mapped (FPKM)” using Cufflinks (ver. 2.2.1). Statistical significance was calculated by comparison between the populations using the Cuffdiff module of Cufflinks. Finally, DEGs passing the significance threshold were identified based on fold change (FC ≥ 1.5 or ≤ 0.67), and the false discovery rate, i.e., FDR, was modified with a 0.05% level for the P value (q < 0.05).

### RT-qPCR analyses

For RT-qPCR analyses, RNAs were extracted from heads and thoraces of two female individuals and then digested by DNase as described for RNA-seq. DNase-treated RNA (300 ng) was transcribed using a high-capacity cDNA Reverse Transcription Kit (Applied Biosystems, Waltham, MA, USA) for single-strand cDNA synthesis according to the manufacturer’s instructions.

Six genes [chitinase 8 precursor (*Cht8*), trehalose transporter (*Tret1*), yellow 3 (*Y-3*), insulin-like growth factor-binding protein (*IGF*), neuropeptide F receptor (*NpF*), and gustatory receptor 48 (*Gr48*)] were selected as target genes. Details of these six genes are described in Table S1. Two reference genes [actin (*Act*) and tbp-associated factor (*Tbpaf*)] were examined with the appropriate primer sets^19^. The primer sequences of target genes were designed using Primer 3 Plus (http://www.bioinformatics.nl/cgi-bin/primer3plus/primer3plus.cgi/). Standard curves for efficiency were generated for each target and reference gene (1, 1/5, 1/10, 1/100 dilutions). The template for these curves was obtained from cDNAs. The relative quantification of cDNAs was performed using a KAPA SYBR FAST qPCR Kit (KAPA Biosystems, Nippon Genetics, Tokyo, Japan) with an RT‒qPCR system (Eco, Illumina, San Diego, CA, USA). Each reaction mixture (total volume 20 μl) consisted of 10 μl of KAPA SYBR Universal qPCR mix, 0.4 μl of each of the forward and reverse primers (10 μM), 7.2 μl of RNase-free water, and 2 μl of the cDNA template. The temperature profile for amplifying the target gene and reference gene fragments was 95 °C for 1 min, followed by 40 cycles of 95 °C for 3 s and 60 °C for 20 s. Each run from an individual sample was repeated three times. The amplification of single products was confirmed by dissociation curve analysis using the RT‒qPCR system.

We recorded the quantification cycle (Cq) number at which each reaction crossed a threshold fluorescence intensity within the linear portion of the amplification curve to estimate the levels of mRNA expression for each gene. The Cq values for *Tbpaf* were stable and not significantly different among the four groups examined (Mann‒Whitney U test, WS1 vs. WL1: z =  − 0.731, P = 0.4647, five samples each; WS3 vs. WL3: z =  − 1.149, P = 0.251, five samples each). Therefore, we confirmed the normalized expression levels for the target genes with the expression levels of *Tbpaf*.

We conducted statistical analysis for these RT‒qPCR data using nested analysis of variance.

## Results

**Figure 2:**
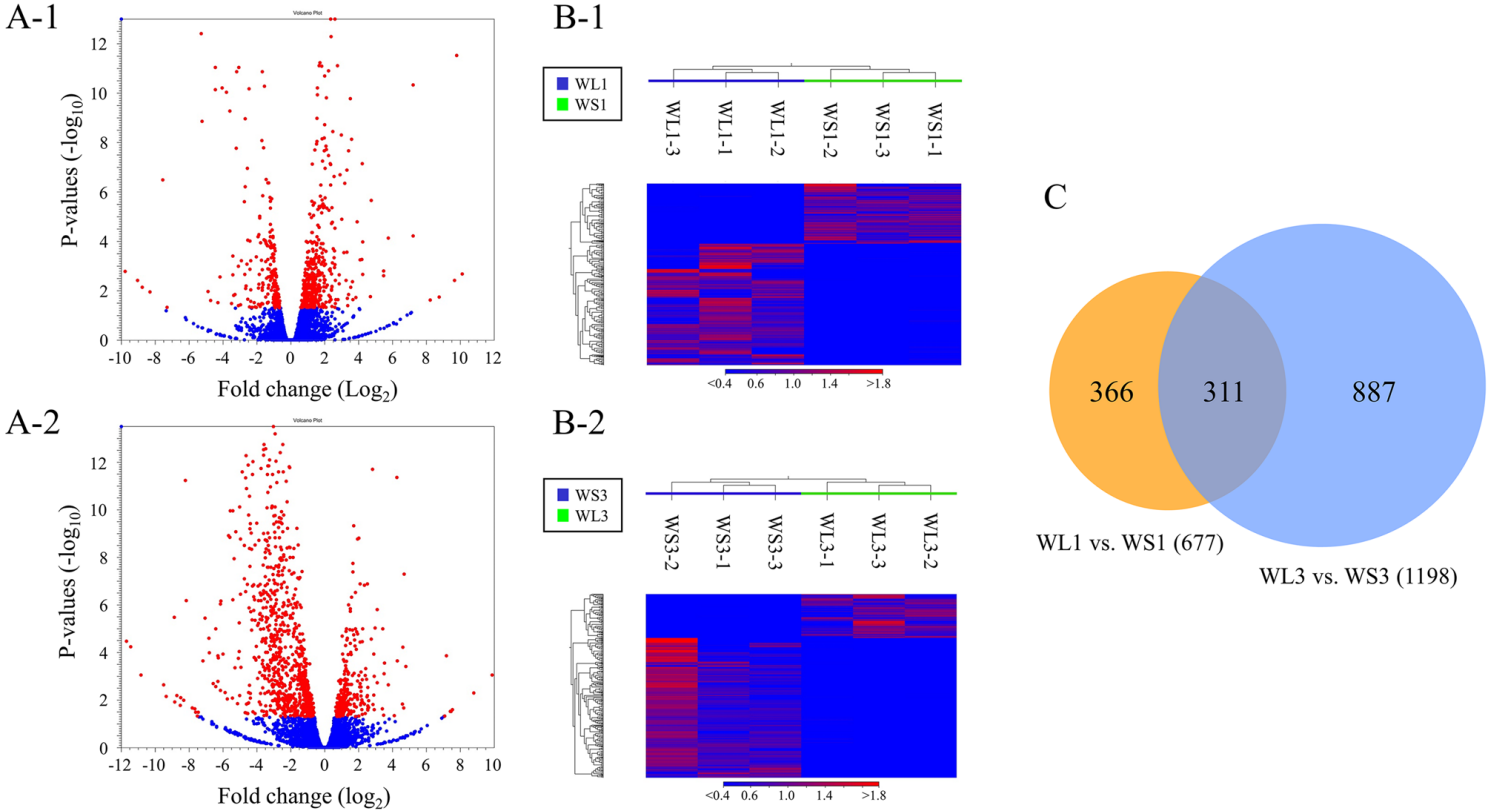
Volcano plot (**A**), heatmap (**B**), and Venn diagram (**C**) comparing the WL and WS populations. (**A**) Volcano plot showing log_2_-fold change (x-axis) and − log_10_ FDR-corrected P value (y-axis) points for all expressed genes in replicate lines 1 (**A-1**) and 3 (**A-2**). (**B**) Heatmap showing gene expression in 3 samples of each population created for replicate lines 1 (**B-1**) and 3 (**B-2**).

Comparison of gene expression levels between the WL and WS populations of *T. castaneum* by RNA-seq analysis revealed DEGs in replicate line 1 (Fig. 2A-1,B-1) and line 3 (Fig. 2A-2,B-2). Genes expressed at higher levels in WS1 (positive log_2_ fold change) were more frequently detected than genes expressed at higher levels in WL1 (negative log_2_ fold change) (Fig. 2A-1), whereas genes expressed at higher levels in WS3 were less frequently detected than genes expressed at higher levels in WL3 (Fig. 2A-2). DEGs were shared among individuals within the populations and clearly separated between the populations (Fig. 2B-1,B-2). DEG analyses revealed 677 DEGs in replicate line 1 and 1198 DEGs in replicate line 3 (Fig. 2C). In addition, 311 DEGs were expressed throughout the replicate lines (Fig. 2C). These DEGs were annotated, and selected DEGs were divided into four main categories: (1) fat- and lipid-related genes, (2) chitin- and cuticle-related genes, (3) sugar- and insulin-related genes, and (4) nervous system-related genes (Tables 1, 2). Nervous system-related genes were more frequently detected in WL populations, some of which encode odorant receptors and the neuropeptide F receptor (Table 2).

**Table 1:**
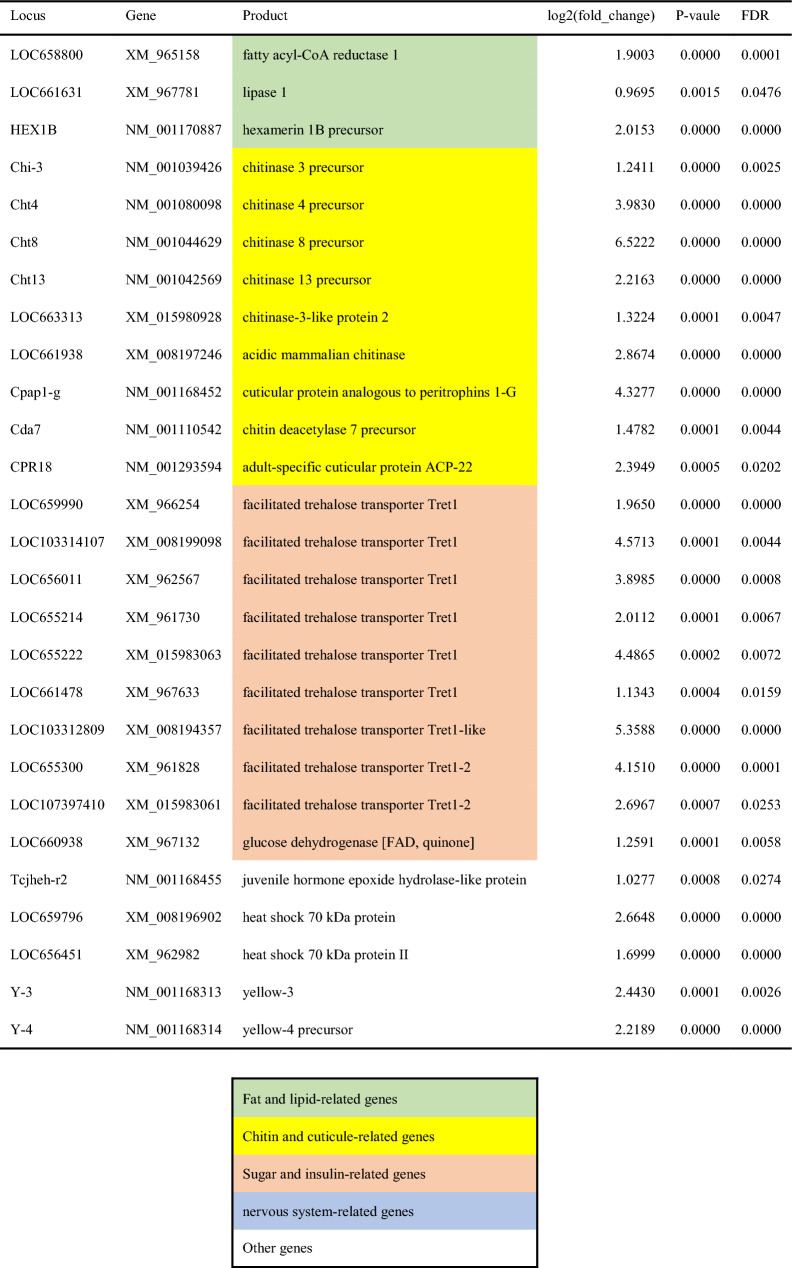
List of selected DEGs whose expression levels were significantly higher in the WS populations.

**Table 2:**
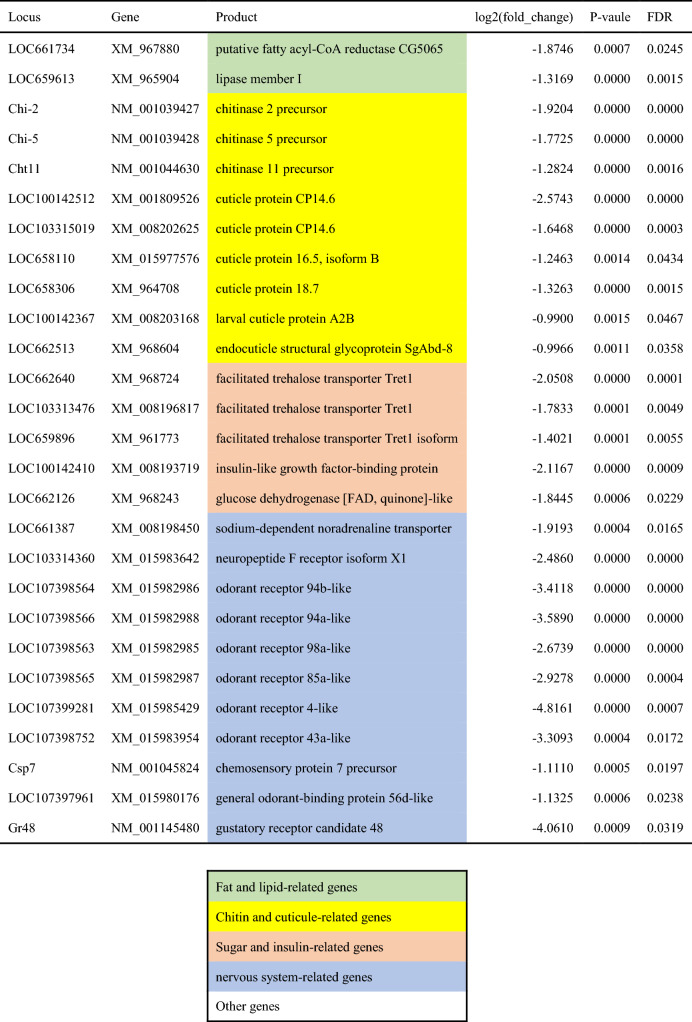
List of selected DEGs whose expression levels were significantly higher in the WL populations.

**Figure 3:**
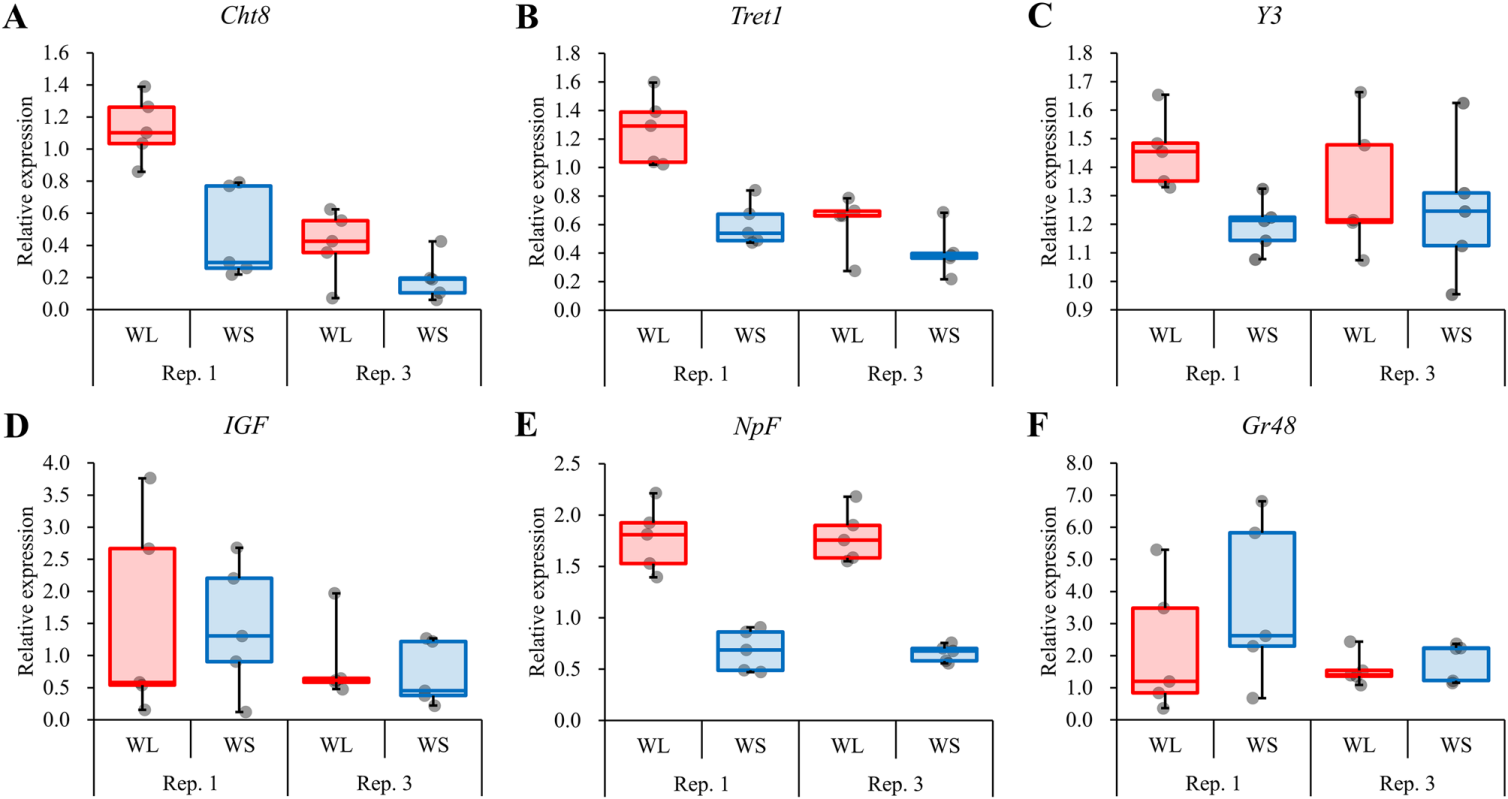
Relative expression of genes (**A**: *Cht8*, **B**: *Tret1*, **C**: *Y-3*, **D**: *IGF*, **E**: *NpF*, **F**: *Gr48*) detected by RT‒qPCR. Red and blue bars show the WL and WS populations, respectively. Error bars show standard errors.

Relative expression levels in some of the common DEGs between lines 1 and 3 were examined by RT‒qPCR. Figure 3 shows the results of RT-qRCR, and the statistical analysis results of these data are shown in Table 3. Among the genes that showed significant differences according to RNA-seq, chitinase 8 precursor gene (*Ch8*), trehalose transporter Tret1 gene (*Tret1*), and yellow-3 gene (*Y3*) were expressed at higher levels in the WS lineage, whereas insulin-like growth factor-binding protein gene (*IGF*), neuropeptide F receptor gene (*NpF*), and gustatory receptor candidate 48 gene (*Gr48*) were expressed at higher levels in the WL lineages. These genes were subjected to RT‒qPCR analysis, and *Cht8*, *Tret1*, and *NpF* showed significant differences between populations (Fig. 3, Table 3).

**Table 3:** Results of nested ANOVA for RT‒qPCR data.

Gene	Factor	*d.f.*	*F*	*P*
*Cht8*	Population	1	20.01	**0.0004**
	Replicate[population]	2	15.61	**0.0002**
	Error	16		
*Tret1*	Population	1	25.18	**0.0001**
	Replicate[population]	2	15.37	**0.0002**
	Error	16		
*Y3*	Population	1	3.88	0.0665
	Replicate[population]	2	0.67	0.5246
	Error	16		
*IGF*	Population	1	0.07	0.7900
	Replicate[population]	2	1.20	0.3263
	Error	16		
*NpF*	Population	1	113.54	** < 0.0001**
	Replicate[population]	2	0.03	0.9719
	Error	16		
*Gr48*	Population	1	1.23	0.2843
	Replicate[population]	2	1.60	0.2331
	Error	16		

Significant values are in bold.

## Discussion

A comparison of gene expression levels between WL and WS populations of *T. castaneum*, which differ genetically in their movement, revealed that 677 genes in replicate line 1 and 1198 genes in replicate line 3 from the artificial selection regime were significantly different in expression between the populations. There were 311 genes that differed in both replicate lines of the selection regime, strongly suggesting that these 311 genes control the movement of the red flour beetle.

Differences between populations were observed for chitin and cuticle-related genes. A previous study showed that superoxide dismutase (SOD) gene knockdown in *T. castaneum* resulted in slower walking and showed knockdown effects in cuticles^23^. In the present study, since the selection was multiplied by walking movement, it is possible that mobility and the cuticle may be genetically correlated, and therefore, differences in gene expression levels may have been observed. Stress tolerance to continuous vibratory stimuli among populations genetically different in death-feigning behavior was found to be lower in terms of survival in populations that exhibited death-feigning behavior for longer durations than in populations that did not^24^. This suggests that exoskeletal robustness is correlated with death-feigning behavior. Since death-feigning behavior is genetically correlated with mobility in *T. castaneum*, this suggests that, similarly, gene expression governing the exoskeleton may also be associated with movement.

Insulin signaling pathways are nutrient-sensing and growth-regulating signaling cascades and influence polymorphisms within species, life history and longevity^25^. The upregulation of genes involved in in insulin signaling pathways and insulin secretion generally causes prolonged longevity in insects^26,27^. In our results, several insulin signaling-related genes were expressed more in the WS population than in the WL population (Tables 1, 2). These results predict prolongation of longevity in the WS population. It is necessary to examine the longevity of both population in the future. A previous study revealed that beetles from the WS population exhibited a significantly longer duration of death-feigning behavior than WL beetles, suggesting that the relationship between movement and death-feigning duration is driven by a negative genetic correlation^28^. In a previous study using selected populations established by artificial selection for death-feigning duration, it was found that expression levels of insulin-related genes were significantly higher in populations with shorter death-feigning durations^19^. This result was contrary to expectations because if movement and death-feigning duration were negatively genetically correlated and gene expression levels were similar, the expression levels of insulin-related genes would be higher in the WL population. It will be necessary in the future to conduct investigations to clarify the cause of this.

There were significant differences in the expression levels of nervous system-related genes between selection regimes. In particular, differences between the WL and WS populations were observed in several genes related to olfactory receptors and taste (Tables 1, 2). This species is known to sense aggregation pheromone (4,8-dimethyldecanal: DMD) by olfaction^29^, and the significantly higher expression in the WL population suggests that females with higher mobility have greater olfactory sensitivity. Indeed, a previous study showed that females from WL populations are more attracted than WS females to aggregation pheromones^15^. Future work is needed to examine the response of females with knockdown of these olfactory-related genes to DMD.

Significant differences were also found in neuropeptide F receptor (NPF). NPF is one of the most abundant and widely distributed neuropeptides in the vertebrate central nervous system, which stimulates food consumption, affects blood pressure, induces anxiolytic effects, enhances memory retention, and influences circadian rhythms^30–35^. Some of the neuropeptide signaling pathways that are structurally conserved between mammals and insects have been revealed^36^. Comparison of circadian rhythms among the selected populations used in this study showed no differences in the period and amplitude of circadian rhythms between populations^37^. It is possible that gene action may differ among species, but more work is needed to compare feeding behavior and memory among these populations.

The RT‒qPCR analysis of six genes based on the RNA-seq analysis showed significant differences among the populations for *Cht8*, *Tret1*, and *NpF*, with higher values for *NpF* in the WL population, which was generally consistent with the RNA-seq analysis results. Moreover, similar results were found in replicate lines. Thus, this result suggests that *NpF* plays an important role in the genetic factors controlling movement in *T. castaneum*. Conversely, in *Cht8* and *Tret1*, RNA-seq analysis showed higher values in the WS populations, but RT‒qPCR showed higher values in the WL populations. Since an effect of replicate lines from the selection regime was also observed, the expression mechanism of these genes should be investigated in more detail in the future. Furthermore, we need additional studies that investigate the effect of knockdown by RNA interference experiments on the genes that showed significant differences according to RT-qPCR in this study.

In a previous study, RNA-seq revealed hundreds of genes, including those correlated with respiration and energy metabolism, that were upregulated in long-winged versus short-winged brown planthopper adults, indicating that long-winged adults might require more energy than short-winged adults for flight^38^. In cotton aphids (*Aphis gossypii*), genes associated with flight-reproduction trade-offs were differentially expressed in winged versus wingless morphs according to RNA-seq analysis^39^. In the brown citrus aphid (*Toxoptera citricida*), RNA-seq revealed both lipid and glycogen metabolism-associated genes that were differentially expressed between winged and wingless adults, indicating that these genes might contribute to energy metabolism during aphid wing development^40^. In this study, the expression levels of energy metabolism-related genes did not differ significantly among the selected populations. This difference in results between the present and previous studies may be due to differences between flying and walking. Similar studies focusing on walking in other species are needed.

Furthermore, there is a possibility that the other genes that were expressed differentially in the WL and WS populations may also be indirectly associated with movement activity. Functional validation of the genes identified as candidates in the present transcriptome analysis to relate them to movement activity should be performed in the future.

In this study, we focused our analysis on the head and thorax of beetles, but we may find different results on the legs and abdomen. In the future, analysis focusing on other parts of the beetle will be necessary. *T. castaneum* is often used as a model animal for RNAi and genome editing (e.g., DIPA-CRISPR)^41–43^. It is also necessary to examine the effects of knockdown by RNAi and knockout by genome editing on the genes (especially *NpF*) for which the present study revealed the possibility of controlling the mobility of *T. castaneum*. In a previous study, the amount of biogenic amines such as dopamine was compared between the selected regimes of *T. castaneum*, but no significant difference was found in biogenic amines^28^. Thus, the physiological factors that control the beetle's walking activity remain obscure. Therefore, our results are expected to improve our understanding of the physiological factors that control the *T. castaneum* walking movement.

### Supplementary Information

Supplementary Table S1.

## Data Availability

The datasets generated during and/or analyzed during the current study are available from the corresponding author on reasonable request.
