# Supplementary material for: Transcriptomic comparison between populations selected for higher and lower mobility in the red flour beetle Tribolium castaneum
Source: Sci Rep. 2024 Jan 2;14:67. doi: 10.1038/s41598-023-50923-6 (PMC10762016; doi:10.1038/s41598-023-50923-6)
Supplement: Supplementary file 1 — Supplementary Table S1. [file 41598_2023_50923_MOESM1_ESM.docx]

**Table S1**. Targeted genes and primer sequences in RT‒qPCR analyses with RNA-seq results.

| Gene | Product | name | RT‒qPCR | | RNA-seq results | |  |
| --- | --- | --- | --- | --- | --- | --- | --- |
|  |  |  | Forward primer | Reverse primer | Fold change (log2) | P-value |  |
| NM_001044629 | chitinase 8 precursor | *Cht8* | GGATCATGGGAGTCGGTGAC | TGGATCAGCCCCTCTCTCAA | 6.522213469 | 3.66897E-23 |  |
| XM_008194357 | facilitated trehalose transporter Tret1-like | *Tret1* | AGTTTGTGCCCGAGACCAAA | GTCACTCCCATCGGCAATCA | 5.358828408 | 2.21616E-13 |  |
| NM_001168313 | yellow-3 | *Y3* | ACGGGGAGTTTCGCTTTTGA | CTTCGTGCCCGTGAAGTACT | 2.442961197 | 5.13354E-05 |  |
| XM_008193719 | insulin-like growth factor-binding protein | *IGF* | GCGCAAATCGGAACAGGTTT | GCAGATTGTCGCAGCTGATG | -2.116697479 | 1.5557E-05 |  |
| XM_015983642 | neuropeptide F receptor isoform X1 | *NpF* | CCAACCACATCTCAGCCCAT | CACCGGATTTGAACATGCCG | -2.485993784 | 3.75133E-14 |  |
| NM_001145480 | gustatory receptor candidate 48 | *Gr48* | CGGCTACACTTTGGCCAAATTT | AGAGACCCATCGCTGTGAAG | -4.060981846 | 0.000917326 | |
